# Broadening Benefits and Anticipating Tradeoffs with a Proposed Ecosystem Service Analysis Framework for the US Army Corps of Engineers

**DOI:** 10.1007/s00267-022-01777-7

**Published:** 2023-01-12

**Authors:** Lisa A. Wainger, Elizabeth O. Murray, Charles H. Theiling, Anna M. McMurray, Janet A. Cushing, Shawn B. Komlos, Alfred F. Cofrancesco

**Affiliations:** 1 Chesapeake Biological Laboratory, University of Maryland Center for Environmental Science, Solomons, MD USA; 2 Engineer Research and Development Center, US Army Corps of Engineers, 3909 Halls Ferry Rd., Vicksburg, MS USA; 3 Wildlife Conservation Society, Bronx, NY USA; 4 National Climate Adaptation Science Center, US Geological Survey, Reston, VA USA; 5 Institute for Water Resources, US Army Corps of Engineers, Alexandria, VA USA

**Keywords:** Ecosystem services, Federal decision support, Toxic contaminants, Corps of Engineers, US EPA Superfund, Benefit indicators

## Abstract

Would-be adopters of ecosystem service analysis frameworks might ask, ‘Do such frameworks improve ecosystem service provision or social benefits sufficiently to compensate for any extra effort?’ Here we explore that question by retrospectively applying an ecosystem goods and services (EGS) analysis framework to a large river restoration case study conducted by the US Army Corps of Engineers (USACE) and comparing potential time costs and outcomes of traditional versus EGS-informed planning. USACE analytic methods can have a large influence on which river and wetland restoration projects are implemented in the United States because they affect which projects or project elements are eligible for federal cost-share funding. A new framework is designed for the USACE and is primarily distinguished from current procedures by adding explicit steps to document and compare tradeoffs and complementarity among all affected EGS, rather than the subset that falls within project purposes. Further, it applies economic concepts to transform ecological performance indicators into social benefit indicators, even if changes cannot be valued. We conclude that, for large multi-partner restoration projects like our case study, using the framework provides novel information on social outcomes that could be used to enhance project design, without substantially increasing scoping costs. The primary benefits of using the framework in the case study appeared to stem from early comprehensive identification of stakeholder interests that might have prevented project delays late in the process, and improving the communication of social benefits and how tradeoffs among EGS benefits were weighed during planning.

## Introduction

Ecosystem goods and services (EGS) analysis and accounting frameworks are promoted as a means to improve natural resource decision making (Millenium Ecosystem Assessment 2005; Bateman et al. 2014; Olander et al. 2017). Perceived benefits of using EGS to inform decisions include avoiding, reducing, or mitigating tradeoffs associated with socio-ecological system change (e.g., Bennett et al. 2009), promoting “design with nature” approaches (e.g., Elmqvist et al. 2013), and enabling markets to develop that could reduce costs of environmental legal compliance (Gómez-Baggethun et al. 2010; e.g., Bolte et al. 2011; Scarlett and Boyd 2015). Some case study analyses have suggested that by considering many EGS benefits in project design and location, rather than only one or a few benefits, the total or net project benefits could be increased (e.g., Nelson et al. 2008; Wainger et al. 2010). Although, a limitation on producing multi-benefit outcomes is that some services will never be fully compatible (McShane et al. 2011). Nonetheless, considering a full suite of applicable services should provide an opportunity to generate more benefits than would otherwise have been possible.

Despite positive projections in the early days of EGS assessment, recent research testing the claims that such assessment improves decisions or conservation credit markets finds mixed results (e.g., Howe et al. 2014; Posner et al. 2016; Wang et al. 2020). Knowledge or analysis of EGS does not necessarily translate to improved management of conflicts between private and public benefits nor increase total benefits of resource management (Howe et al. 2014). Further, it is generally understood that economic valuation of EGS to support site-specific decisions may not fully inform decisions (Holmes 2020). Therefore, a critical question for the continued development of EGS frameworks and analysis is, “Do such frameworks improve ecosystem service provision or total social benefits, compared to other decision processes?” Embedded within this question is the understanding that frameworks must be practical and supported by available science, if they are to be implemented effectively.

We address the question of whether EGS analysis increases project net benefits by qualitatively comparing the potential costs versus gains of using an EGS analytic framework, where gains may be higher social benefits or increased management efficiency. We compared how EGS were used in a current design process versus a hypothetical design process that applied the scoping steps of a new classification and analysis framework that we developed to be compatible with the US Army Corps of Engineers (USACE) procedures. We used data and information from existing reports created for a multi-purpose project that was recently planned by the USACE Civil Works Planning, in partnership with US EPA and others.

The USACE decision-making methods are a useful focus for ecosystem service framework development because USACE policy has a strong influence on environmental restoration design in the United States (Reed et al. 2013). Although most people equate navigation and flood control with USACE projects, the Water Resources Development Act (WRDA) of 1986 initiated expansion of USACE’s missions to include improvements of the environment, and a host of similar environmental improvement authorities were created in subsequent WRDAs (National Research Council 2004), including an Aquatic Ecosystem Restoration authority (WRDA 1996). The new authorities enabled the USACE to partner with state and local governments and non-governmental organizations to implement aquatic ecosystem restoration projects to improve environmental conditions.

Recent USACE guidance has added urgency to the issue of developing cost-effective ecosystem service analysis methods by emphasizing the goal to measure a comprehensive range of economic, social, and environmental effects [U.S. Assistant Secretary of the Army (Civil Works) 2021], which will encompass many EGS. Past efforts to consistently measure EGS have been hindered by a lack of common definitions and measures for EGS, within the USACE and elsewhere (Murray et al. 2013; Tazik et al., 2013). Interpretations of EGS outcomes for USACE projects have ranged from objective measures of benefit or harm to people, to basic biophysical changes that represent unspecified benefits (e.g., Bridges et al. 2015; Cushing et al. 2022). Civil Works authorities and policies appear to generally support or allow consideration of EGS information in planning water resources projects, though plan justification requires tying benefits to the project authorities (USACE 2000; Reed et al. 2013).

Although many comprehensive ecosystem service categorization, mapping, and analysis frameworks have been developed for use by national government agencies (e.g., Boyd et al. 2016; DeWitt et al. 2020; Newcomer-Johnson et al. 2020; Haines-Young and Potschin 2013), we did not find one that met the needs of the USACE when the effort to establish an EGS classification and analysis framework began more than ten years ago. A primary concern with some systems in common use was that many EGS accounting systems equate ecosystem service quality and monetary value with land cover and land use, rather than representing biophysical qualities of that land cover and landscape, as might be needed to represent restoration benefits (Martínez-Harms and Balvanera 2012; reviewed in Chan and Satterfield 2020). After reviewing existing options, we concluded that a new framework, tailored to the USACE, would be needed to comprehensively capture relevant EGS, and be consistent with existing planning policies and project authorities. In particular, some EGS are discouraged from being monetized in USACE policy and therefore would need to be classified and analyzed separately from monetized EGS using non-monetary benefit indicators for a policy-compliant version of the framework. These non-monetary benefit indicators (also referred to as benefit relevant indicators, Olander et al. 2018) fill a decision support need by capturing site-specific qualities that reflect relative benefits that can be difficult to monetize (Wainger and Mazzotta 2011). Although they do not directly represent economic benefits, they can be weighted to represent relative values and willingness to make tradeoffs among EGS.

The paper is structured to first describe the Ecosystem Goods and Services Analysis Framework (hereafter the EGS Framework) that we developed to strengthen the representation of social benefits or harms in USACE planning analysis and then evaluate a case study test. This framework is distinct in how it defines and classifies ecosystem services and the analytic methods have been tailored to the USACE. However, it shares some concepts and approaches with other ecosystem service framework efforts developed to support US federal agencies in 2013–2015 and earlier, as a result of collaborative idea development. Perhaps the most similar framework to this one is the Federal Resource Management and Ecosystem Services Guidebook (NESP 2014), that multiple authors on this paper contributed to (Wainger was a co-author; Cofrancesco, Cushing, Komlos and Murray were advisors). The classification system used here is simpler than ecosystem service classification systems developed by and for the US EPA (e.g., DeWitt et al. 2020; Newcomer-Johnson et al. 2020) but shares some concepts, in part, due to advisory work for the US EPA Ecosystem Services Research Program and earlier supporting work by Wainger (e.g., Boyd and Wainger 2002; Munns et al. 2015; Wainger and Mazzotta 2011; Wainger et al. 2010).

The methods section describes the main analysis elements of the EGS Framework, with full details available in Wainger et al. (2020). The results section provides details of the test implementation for a multi-objective river restoration project study in the Meramec River, Missouri (USA). The Discussion section summarizes our evaluation of the analytic burden and potential benefits of using the EGS Framework, relative to current planning methods by addressing the following questions:

1. Does EGS planning cost more than current USACE planning?
2. Are EGS benefits likely to increase as a result of the EGS Framework implementation?
3. Is adopting the framework likely to generate net benefits?

## Methods for Ecosystem Service Analysis Tailored to USACE

The EGS Framework is a set of techniques and tools for rapidly characterizing EGS benefits or harms, institutional priorities, and tradeoffs of alternative projects or project designs. The main steps are to identify the set of potential EGS that are most relevant to maximizing benefits through project design and choose appropriate indicators and measurement approaches for EGS benefits and harms of a project. The framework adds to existing methods in three primary ways. First, it leads the analytic team to screen potential project effects early in project planning, using a broad set of EGS effects, regardless of the specific project mission areas or goals (called authorized project purposes) that are specified for restoration projects when Congress authorizes project budgets. Second, it uses response functions to evaluate whether biophysical changes are meaningful in terms of overcoming existing levels of degradation and substantially improving human benefits. Third, it proposes quantitative indicators of total potential use, scarcity or substitutability of an EGS, to reflect the relative value of improvements at a site. These three elements are reflected to some degree in existing methods, but are not uniformly applied.

The use of scarcity in assessing potential benefits of changes from a project embeds the fundamental economic principle of ‘diminishing marginal utility’ (Freeman et al. 2014) that says that people tend to gain lower benefits from consuming additional units of a good, once their initial needs or wants have been satisfied. Similarly, many governmental acts (e.g., US Endangered Species Act) serve to prioritize spending to increase or prevent a decrease in a scarce public good or service. Although exceptions to this principle are possible, we apply it in the framework as a generally useful, but not absolute, prioritization rule.

### Defining EGS

The team defined EGS as, “socially valued aspects or outputs of ecosystems that depend on self-regulating or managed ecosystem structures and processes” (Murray et al. 2013) to clarify two concepts. First, a change in ecosystem aspects or outputs must be valued by people. They must be able to determine whether they would benefit or be harmed by a change in the amount or quality of the thing being measured. Second, EGS must be derived from ecological systems, and not fully engineered systems, although systems may include elements not found in nature (e.g., dikes and pumps) that enable the ecosystem to deliver services. Hence, EGS represent a subset of beneficial services that might be provided by a USACE project, since built or “grey” infrastructure are often used to provide desired public services, such as flood control.

The term *socially valued* includes all tangible and intangible EGS that are used or appreciated. The tangible benefits, or use services, such as recreation and flood risk mitigation, produce highly visible benefits to people. The intangible, or what we call nonuse or passive use *services* (but are more commonly referred to as nonuse or passive use *values*), are derived from widely held feelings of stewardship towards the environment, including concern for other people who may depend on or simply enjoy ecosystems now or in the future (details in Wainger et al. 2018).

To develop EGS that are valued by people, basic biophysical information is contextualized in terms of improvements in well-being. Without context, a biophysical indicator often will not represent direction and magnitude of benefits. For example, sediment accretion alone can have a positive, negative, or neutral effect, depending on whether deposition is occurring on a protective barrier island, in a navigation channel, or in an offshore site. Some authors have referred to this distinction between biophysical inputs and service outputs or outcomes as *intermediate* and *final* services and describe the advantage that such a system has in avoiding double-counting of benefits (Boyd and Banzhaf 2007; Fisher et al. 2009; Wainger and Mazzotta 2011; Johnston and Russell 2011). Using this distinction, we exclude “supporting” and some of the “regulating” ecosystem services from the Millennium Ecosystem Assessment typology of services (2005) that serve as inputs or intermediate services and generally use more parsimonious categories, compared to other federal ecosystem service frameworks.

*Self-regulating* is a goal that the USACE has historically applied to ecological restoration projects and is defined as systems that do not require human technology or intervention to produce benefits. However, our EGS definition modifies that historical goal to acknowledge that many ecosystems require novel elements or ongoing management to maintain benefits in human-dominated landscapes, where stressors may overwhelm a restored ecosystem (Seastedt et al. 2008; Hobbs et al. 2009). The point at which a restored ecosystem shifts from mostly natural and capable of generating ecosystem services to one that is mostly engineered and therefore provides beneficial services, but not ecosystem services, is not easily defined and most projects fall between the two extremes. However, it is important to distinguish goods and services derived from soil, plants, animals and all living organisms versus human-built elements because to say that an engineered concrete structure alone generates ecosystem services implies that engineered systems are fully substitutable for natural systems.

Through a series of workshops and conversations with USACE scientists and stakeholders, we developed 12 general non-overlapping ecosystem service categories (Table 1) for analysts to use to organize detailed and site-specific EGS. Two EGS that are commonly lumped, water supply and water quality, are separated because cases can arise where only one is authorized for a project. The list includes some intermediate EGS (e.g., carbon sequestration), when measurement of the final EGS would be intractable.

**Table 1:** Definitions and examples of ecosystem goods and services defined for USACE (modified from Wainger et al. 2020)

Category	Definition	Example EGS Indicators^a^
Ecosystem Sustainability (Nonuse and passive use EGS)	The maintenance of ecosystems’ structural and functional qualities to secure species and ecosystems and promote resilience. Includes all nonuse or passive use services (existence, intergeneration bequest, or altruistic values) derived from the condition of species or ecosystems.	• Native species diversity/other indices of ecological community condition & maintenance of future viability • Population viability of a threatened species • Frequency of stream base flow meeting an ecologically relevant threshold (e.g., required to maintain a fish population)
Water Supply & Regulation	The provision and regulation of surface and groundwater by ecosystems that may be used for domestic, municipal, industrial, and agricultural purposes.	• Recharge rate of a water supply aquifer suffering declining levels • Change in hydropower output
Hazard Mitigation (Reduced risks to Property & Infrastructure, Human Safety)	Ecosystem-induced reduction of risk or vulnerability to natural hazards that threaten property, infrastructure, human safety, or natural resources. Threats include storms, floods, landslides, wildfires, and droughts.	• Residential and commercial property protected by flooding due to storm surges • Bushels of corn protected from inland flooding
Navigation Maintenance	Provision of safe and efficient waterborne transport of goods and people as supported by sediment reduction and water regulation by functioning ecosystems^b^.	• Sediment accumulation rate in navigable channels (e.g., as a function of land management) • % channels with levels of non-native aquatic growth compatible with vessel operation
Recreation Supply	Quantity and quality of recreational opportunities.	• Catch per angler • Number and/or diversity of ecotourism activities supported
Cultural, Spiritual & Educational Support	Maintenance of opportunities arising from sites and landscapes that have spiritual or religious significance, contribute to a “sense of place”, or sustain cultural heritage, including traditional ways of life. Also includes opportunities for scientific discovery and education.	• % buffering of documented historic, cultural or religious sites with open space or natural vegetation • Acreage of relatively undisturbed ecosystems, accessible for research
Aesthetics	Aesthetic enjoyment provided by the condition and relative placement of landscape and ecosystem features, pleasing to one or more of the five senses. This is typically an intermediate service contributing to property value enhancement, recreation, and cultural or spiritual values.	• % of view from residences or sensitive commercial properties that is open water • Frequency of lake turnover events emitting sulfur smell • Forest clearcuts observable from road
Food Provisioning	Provisioning of or contribution to commercial or subsistence production of food and the ecosystem conditions that support it.	• Abundance of a fish species used for food within tribal communities • Population density of edible fish in streams/lakes used by fishers
Raw Goods & Materials Provisioning	Provisioning of or contribution to raw goods and materials.	• % accessible sand with grain size suitable for construction uses • Growth rate of harvestable trees
Water Purification & Waste Treatment to Protect Human Health^c^	Filtration and removal of excess nutrients or pollutants by ecosystems from inland, coastal, or marine waters.	• Pathogen concentration in water adjacent to public beaches • Frequency or extent of harmful algal blooms in areas of commercial fishing use or recreation
Climate Regulation, Carbon Sequestration	Ecosystem moderation of adverse climate effects through sequestration of greenhouse gases.	• Annual carbon sequestration rate of restored forest
Human Health Support (other than water purification)	Ecosystem reduction of the risk of or vulnerability to health hazards other than water quality. Includes changes in air quality, environmental stressors, and animal or insect disease vectors.	• Days of unhealthy levels of particulate matter • Frequency of urban temperatures considered dangerous to health • West Nile disease frequency

^a^Not all example indicators would be used by USACE to formulate or select plans but may be important to stakeholders

^b^Built structures such as levees, dams and seawalls are not considered to provide ecosystem services but they contribute indirectly to the ecosystem service of navigation maintenance since they can help to maintain the wetland and forest ecosystems that will prevent sediment from blocking navigation

^c^Water purification for human health is separated from related EGS in order to provide the ability to distinguish when water quality improvements can be considered to fall within a USACE mission area vs when they are being tracked to address stakeholder concerns or NEPA requirements. Water quality improvements that benefit species or ecosystems may be captured under Ecosystem Sustainability and Recreation

### Overview of Proposed USACE EGS Framework

**Fig. 1:**
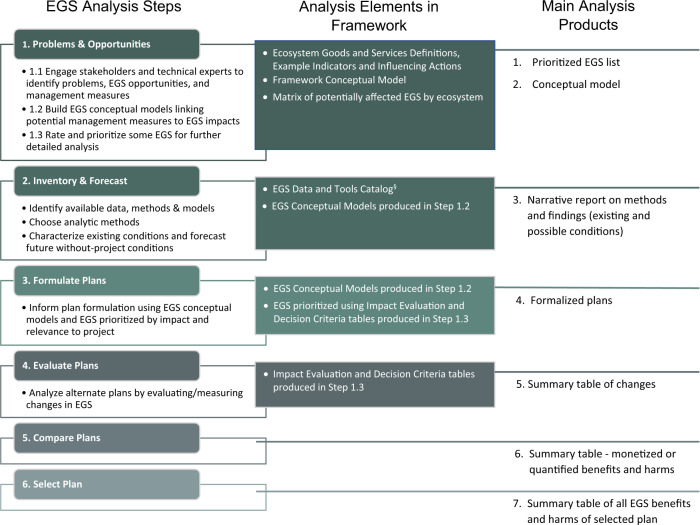
Overview of the integration of EGS analysis framework and six-step planning process. Major headings in Column 1 outline the current USACE 6-step planning process and bullet points show EGS Framework elements (some of which overlap with current methods). Columns 2 and 3 show new analytic elements and outputs proposed in the EGS Framework

The proposed USACE EGS Framework builds on the traditional USACE “6-step” planning process (USACE 2000) by developing a systematic approach to evaluating and communicating the EGS that are potentially influenced by a project (Fig. 1). Given current policy restrictions, this comprehensive EGS Framework could be used for screening project alternatives but not used in selecting the final plan. The method also has the potential to streamline environmental evaluations that are often done separately during planning, such as environmental impact statements created for compliance with the National Environmental Policy Act (NEPA).

#### Causal chain conceptual models for identifying EGS effects

At the core of the EGS Framework is the *causal chain model*, which is a conceptual model used to rapidly identify the EGS that are potentially affected by a project and the information needed to quantify effects (defined in Olander et al. 2018 and Wainger and Mazzotta 2011). Relationships are constructed in diagrams to document how proposed management actions are expected to generate biophysical changes and then socio-economic changes (benefits or harms). The relationships are constructed using established science and the types of preliminary research that are typically conducted as part of initial project planning.

**Fig. 2:**
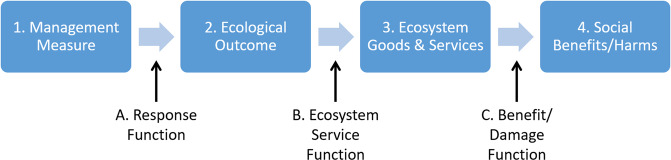
EGS causal chain model. The conceptual model includes three main steps to evaluate potential social benefits or harms of a proposed project. The response function links management measures to changes in biophysical outcomes that are recognizably important to people. The ecosystem goods and services evaluation characterizes the magnitude of potential benefit from ecological outcomes using the number of potential beneficiaries or level of conservation concern. The benefit/damage function measures magnitude of concern among beneficiaries using monetary valuation or preference-weighted indicators

The causal chain model consists of four main conditions (numbered boxes in Fig. 2) connected by three relationships (lettered arrows in Fig. 2). The first condition is the set of management measures, or proposed actions that modify an ecosystem. These actions are connected to the second condition, the set of ecological outcomes, or the “socially valued aspects or outputs of ecosystems”. Ecological outcomes are then linked to EGS (potential) benefit indicators representing evidence of human concern, and then connected to social values that measure the strength of preferences for the EGS. More details and examples of conditions and relationship types are provided in Results.

The horizontal arrows in Fig. 2 are relationships that describe the magnitude of responses to changes in the first condition in terms of changes in the second condition. The first arrow, the response function (Arrow A, Fig. 2), specifies the direction and magnitude of expected biophysical changes as a function of the management effort, in the context of other conditions. For example, a wetland’s size and design characteristics could be linked to the expected change in the quantity and quality of improved animal habitat.

The second arrow in Fig. 2, ecosystem service evaluation (Arrow B), characterizes the evidence that ecological outcomes have the capability of generating human benefits (Box 3, Fig. 2). Two types of evidence are incorporated at the screening stage, the number of potential beneficiaries and the scarcity of the service. Number of beneficiaries is applied in the case of use services only since nonuse values can accrue to beneficiaries who are quite distant from the site, making their measurement difficult. For nonuse services, the degree of conservation concern and relative availability of alternative potential restoration sites would indicate benefit magnitude.

The third arrow, the benefit or damage function (Arrow C, Fig. 2) represents evidence for the magnitude of social benefits/damages. Benefits or damages are derived from both the change in the ecological outcome and the change in amount of human use, enjoyment, or satisfaction. At the full analysis stage, these benefits or harms could be measured as the willingness of people to give up other goods and services to increase the specified ecosystem good or service, as measured in monetary units (Freeman et al. 2014; Johnston et al. 2015) or as preference-weighted indicators within a multi-criteria decision framework (Keeney 1982; Davies et al. 2013). The social vulnerability of the population served might also be used in evaluating the magnitude of benefits in terms of distributional effects (see Rufat et al. 2019). For nonuse EGS, the rank of conservation priority, under Box 3, would typically be used to measure the degree of conservation concern.

The causal chain modeling and analysis results are distilled into two summary tables. The *Impact Evaluation Table* is used to summarize and communicate the potential magnitude of EGS impacts and benefits (shown in results). *The Decision Criteria Table*, summarizes whether EGS goals are consistent with policy and provide documentation of assumptions and sources of risk (shown in Wainger et al. 2020).

#### Impact evaluation table

The Impact Evaluation Table essentially provides an initial risk or benefit assessment of ecological changes, represented as an aggregate impact rating. The rating jointly characterizes the size, duration, and social consequences of each EGS change. Three main criteria are used to assess whether effects are substantial (1) biophysical effects - magnitude and duration of ecological change, (2) beneficiary effects - magnitude and duration of effects on people or a resource of concern, and (3) substitutability - availability of substitute sites that can produce the service.

Determining whether biophysical or beneficiary effects are substantial does not follow hard and fast rules in federal decision making, even when outcomes are regulated (e.g., Van Houtven and Cropper 1996). A biophysical change may be deemed substantial either because it moves an ecosystem element across a quality threshold to produce (or eliminate) an EGS, or otherwise improves/degrades system function to a degree deemed meaningful by subject matter experts and concerned stakeholders. For example, a stream’s trout habitat conditions (e.g., temperature and fine sediment concentrations) could be modified to the extent that the stream gained the capacity to support trout reproduction. Temporary and short-term impacts are generally less important than long-term impacts, but determinations can vary by project (Table 2). Whether a beneficiary impact is substantial is based on the relative number of users and/or the expected level of concern about the change, as identified through stakeholder engagement and simple analyses.

**Table 2:** General criteria for determining substantial impact based on magnitude and duration of effect

		Duration of Effect		
		Temporary	Short-term	Long-term
Ratings for biophysical or beneficiary impacts	Negligible	NO	NO	NO
	Small magnitude	NO	NO	MAYBE*
	Medium magnitude	NO	MAYBE*	YES
	Large magnitude	MAYBE*	YES	YES

*Determinations made on a case-by-case basis. Definitions for table terms in Wainger et al. (2020)

The final ranking in the Impact Evaluation Table is substitutability. Although people may adapt to the loss of an EGS by substituting a completely different service, we assume that wellbeing declines as EGS availability declines, an assumption that may overestimate welfare losses (Rosenthal 1987). The substitutability of services provided onsite, such as recreation services, can be evaluated by identifying the number of comparable sites serving the same population (e.g., USACE 2000 App E, Section VII). Services provided in proximity to a site, such as flood risk mitigation, might be evaluated in terms of relative risk reductions.

For nonuse services, the current and future availability of alternative restoration locations (or lack thereof) will affect substitutability. For example, knowing that other available habitat restoration sites are overrun with a difficult to control and harmful non-native invasive species can elevate the importance of restoring an uninvaded site. A wealth of existing research and stakeholder knowledge supports the ability to identify scarcity of nonuse environmental values in terms of existing habitat (e.g., The Nature Conservancy 2011), potential habitat, and the minimum area and connectivity of habitat types for sustaining species (Myers et al. 2000; Knight et al. 2008; Kocovsky et al. 2009; Wilson et al. 2011; Mappin et al. 2019; examples include Hamilton et al. 2022). To be compatible with the framework, scarcity/substitutability measures must only reflect conservation priorities and not be lumped with other decision criteria, such as land cost, to avoid double-counting criteria that are used later in the decision process.

**Fig. 3:**
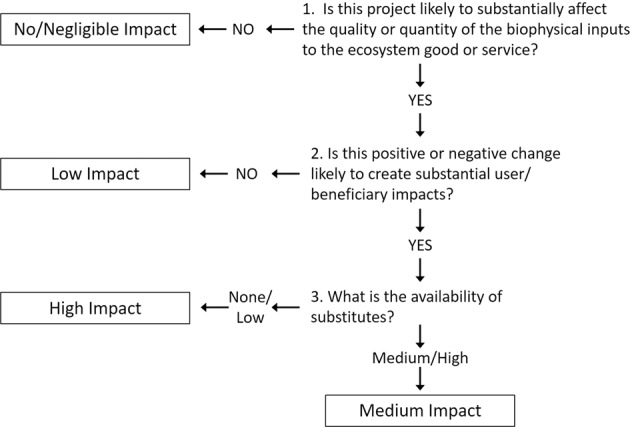
Decision tree for assigning aggregate impact rating to an EGS change

After making the determinations of whether ecological and beneficiary impacts are substantial (using Table 2) and assessing scarcity, analysts combine these criteria to generate an aggregate impact rating in the Impact Evaluation Table, using the decision tree in Fig. 3. (Results show illustration of methods). Table 2 and Fig. 3 provide guidance and add transparency to EGS screening judgments (i.e., whether EGS are included/excluded in later analysis) compared to current processes that do not always document underlying rationale. The intention is that stakeholders can review this guidance and be given the opportunity to debate these criteria for a given EGS and project. EGS that are rated as having high or medium impact at this stage are the most likely candidates for use in formulating project designs (plan formulation) or in a benefit assessment of a recommended plan.

### Case Study Test of the EGS Framework

**Fig. 4:**
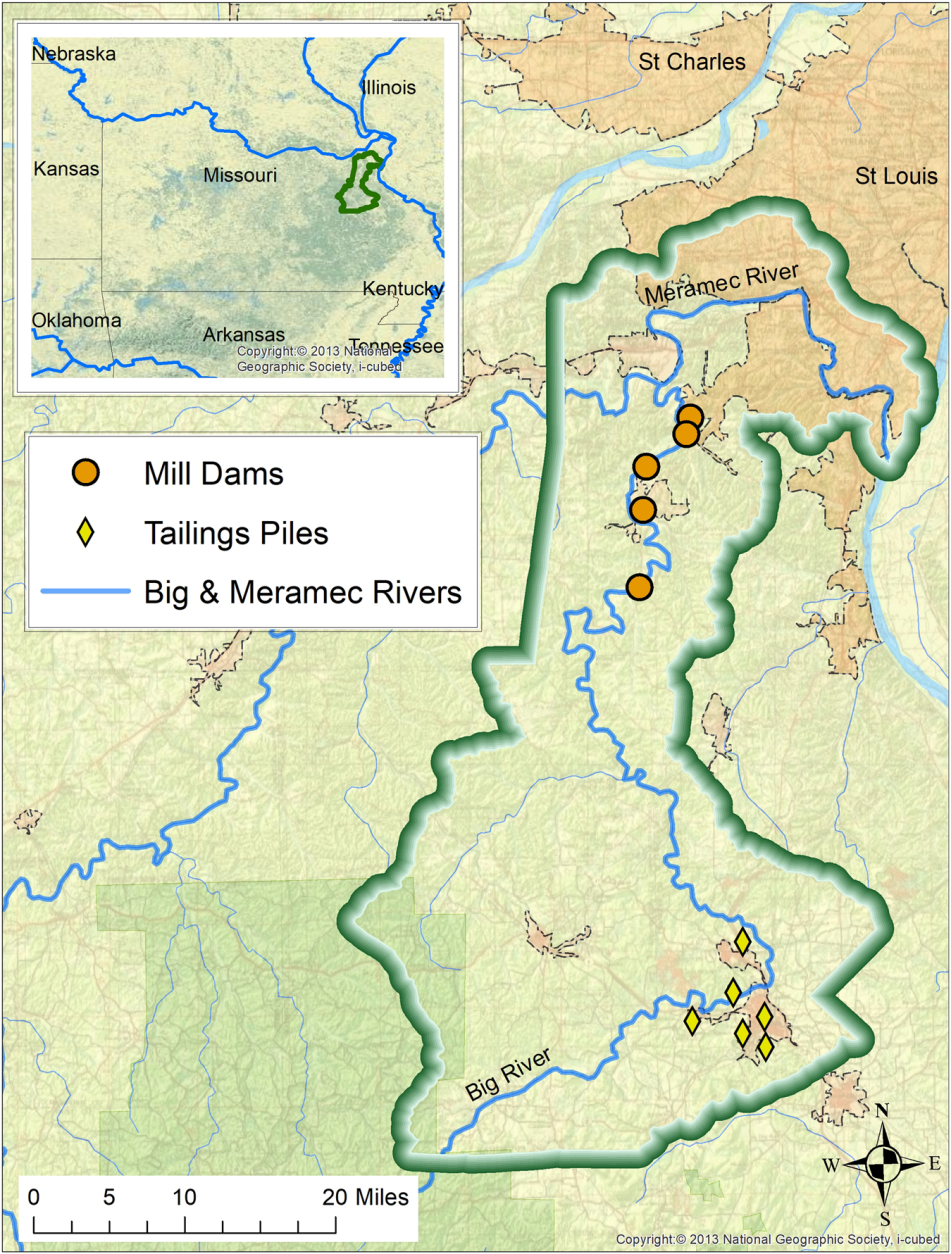
Map of Meramec River Case Study. The study area includes the Big River and the lower 50 river miles of the Meramec River, which is a tributary of the Mississippi River. Source data and information from (USACE St. Louis District 2019). Figure uses ArcGIS online base map provided by National Geographic Society and was created using ArcGIS® software

We tested the proposed EGS Framework’s potential net benefits using a case study analysis of the St. Louis Riverfront - Meramec River Basin contaminant remediation and ecological restoration (Fig. 4). This study was selected because of the availability of high-quality information and the anticipated challenges associated with the integration of US EPA Superfund and USACE program planning. The US EPA initially designed a lead contamination remediation plan for the Big River, which is a tributary of the Meramec River, to fulfill Superfund cleanup goals for protecting human health and the environment (US EPA Region 7 2018). The USACE was subsequently engaged in planning to support ecological restoration goals. The US Fish and Wildlife Service, Missouri Department of Natural Resources, and other governmental and non-governmental organizations were also involved in planning.

Our EGS Framework test used existing published reports, presentations, interviews with project analysts, and reviews by project managers and expert judgment to qualitatively compare interim project decisions with outcomes of a screening-level EGS Framework analysis (Planning Step 1). No new data or analyses were used and some inputs were from draft products. Therefore, results should be considered illustrative only.

The restoration project aims to ameliorate effects of past mining activities in the watershed by reducing the downstream migration of contaminated sediment, currently held in tailing piles and behind mill dams. According to the US EPA, the heavy metal contamination (lead, zinc, and cadmium) creates risks to human health, aquatic ecosystems, and terrestrial species (US EPA Region 7 2018). Ecological restoration goals include protecting and enhancing aquatic and riparian ecosystems. The Meramec River is thought to contain “one of the most diverse unionid mussel faunas in the central United States with >40 species identified” (Hinck et al. 2012) and four endangered mussel species are thought to occur in the study area (USACE St. Louis District 2019, Appendix A, p. A-27) and all of the species present are vulnerable (USACE Meramec FS Final report, Tables 2–3). Terrestrial species that eat invertebrates that concentrate the metals from the sediment are at risk because they are exposed to levels of heavy metals (e.g., Kingfishers, American Woodcock) that exceed safe levels, which EPA defines as no detectable health effects (US EPA Region 7 2018).

**Table 3:** Management measure screening criteria (from USACE, St Louis 2019)

OBJECTIVES	MANAGEMENT MEASURE	RETAINED FOR EVALUATION	SCREENING CRITERIA/DESIGN CONSIDERATIONS
Riparian Corridor	Reforestation	Yes	Meets all criteria, retained
	BMPs	No	Recommended as Non-Federal Sponsor activity
Emulate a Natural and Stable River	Gabion baskets	No	High cost (not efficient due to higher costs than other measures) and not environmentally acceptable
	Rock riprap along surface (revetment)	No	High cost (not efficient due to higher costs than other measures)
	Bendway weirs	Yes	Meets all criteria, retained
	Stream Barbs	Yes	Meets all criteria, retained
	Longitudinal peak stone toe protection (LPTSP)	Yes	Meets all criteria, retained
	Concrete channelization	No	High cost (not efficient due to higher costs than other measures) and environmentally unacceptable
	Reshape bank	Yes	Meets all criteria, retained
	Root wad revetment	Yes	Meets all criteria, retained
	Live plantings	Yes	Meets all criteria, retained
	Tree revetment	Yes	Meets all criteria, retained
Reduce Sediment	Sediment basin	Yes	Meets all criteria, retained
	Install new low head dam	No	High cost (not efficient due to higher costs than other measures) and environmentally unacceptable
	In-stream excavation	Yes	Meets all criteria, retained
	Grade control structure	Yes	Retained for bed stability
	Side channel with sediment trap	No	Similar to sediment basin
	Bed sediment collector	Yes	Meets all criteria, retained
	Conservation easements	No	Recommended as non-federal sponsor activity

Congress authorized the USACE to address “public access, navigation, harbor safety, off-channel fleeting, intermodal facilities, water quality, environmental restoration and protection, and related purposes” (USACE St. Louis District 2019 Appendix A) and USACE chose to address the ecosystem restoration portion of the authorities as most relevant and beneficial. Other partners wanted to improve a wide array of EGS provided by the Meramec River and watershed, including supporting water-based recreation to local residents, farming, reducing flood risk, and reducing stress on habitat for species at risk (The Nature Conservancy 2014; US EPA 2014; USACE St. Louis District 2019 Appendices).

## Meramec River Case Study Results

We compared the decision steps and outcomes of the current planning process to a hypothetical alternative process that applied the initial steps of the EGS Framework for the Meramec Case Study. For each of the three EGS Framework elements in Planning Step 1 (Fig. 1), we considered whether using the new EGS Framework guidance might have altered planning outcomes. The discussion section that follows uses the results to address the questions raised in the introduction about the relative costs and benefits of adopting the EGS Framework.

Our depiction of the existing planning process used is not an exact representation because it would be difficult and repetitive to represent the multiple phases of decision making. This example primarily uses results from the decision support process developed after the USACE was involved up through an interim Record of Decision (US EPA Region 7 2018) that has since been retracted due to stakeholder concerns (US EPA Region 7 2019). It adds some project elements and outcomes from earlier phases conducted solely by US EPA, for completeness.

### Step 1.1. Identify Problems, EGS Opportunities, and Management Measures

A USACE-led planning team identified ecosystem management measures and gaged their effectiveness at addressing concerns for endangered mussels and aquatic and terrestrial habitat. The three primary USACE restoration goals were (1) Reduce the downstream migration of excess mining-derived sediment from the Big River, in order to protect and restore degraded aquatic and freshwater mussel habitat; (2) Restore impacted channels and floodplains in the Big River and Meramec River systems to mimic a more natural, stable river; and (3) Increase riparian habitat connectivity, quantity, diversity, and complexity (USACE St. Louis District 2019, p. 56). In a separate decision process, the EPA simultaneously developed options for contaminant remediation practices to minimize risk to people and ecosystems in the Big River including sediment excavation and stabilization, and monitored natural recovery (allowing contaminants to be buried or flushed without major human intervention, but using adaptive management), among other practices (US EPA Region 7 2018). Earlier phases of the EPA-led remediation project had already installed a check dam (Newberry Riffle), reinforced the Rockford Beach Dam with rock, and taken some remediation steps to prevent movement of mine tailings and prevent exposure on private property (US EPA Region 7 2011).

The structure of the planning steps within the EGS Framework are similar to traditional planning but an important difference is whether human benefits are implicit or explicit when the team analyzes and communicates internally about the ability of project design elements to meet objectives. Table 3 reveals how the restoration options (management measures) were screened and communicated within the USACE-led planning team. The objectives in Table 3 (column 1) represent ecological features and functions that implicitly represent potential human benefits. For example, the term “Riparian Corridor” has the potential to confer many types of benefits. By reading other documentation, the goals associated with this objective were clarified as minimizing human exposure to contaminants and improving aquatic habitat. The remaining columns show which design features were retained, shown as “yes” in the column “retained for evaluation” (Column 3), and the justification for rejecting design features (Column 4). An earlier version of the table included additional measures, including wetland creation under Riparian corridor objective, that was rejected with “Not efficient, does not meet study objective” a decision that was later questioned by stakeholders (see Discussion).

By not specifying the likely EGS effects of design choices at this initial screening stage of project development, the process does not document how participants reached conclusions about the potential magnitude of benefits of alternative management measures. Also missing from Table 3 are many ancillary benefits or harms such as recreational boating and fishing, shore-based recreation, drinking water protection, and dredging costs avoided. These potential benefits were explored at some point in the project analysis (USACE St. Louis District 2019 Appendices), however, the available documents do not demonstrate how or whether these goals were used in project formulation.

If the EGS Framework were followed, Table 3 would have explicitly listed the affected EGS and diverse stakeholder concerns. This information would then be carried through to Step 1.2 where any tradeoffs that were made among goals (internal and external to project authorities) would be documented and acknowledged. Details of how all these potential benefits, harms, or tradeoffs were applied during design choices will be shown, in detail, in Step 1.3.

### Step 1.2 Build Causal Chain Conceptual Models

**Fig. 5:**
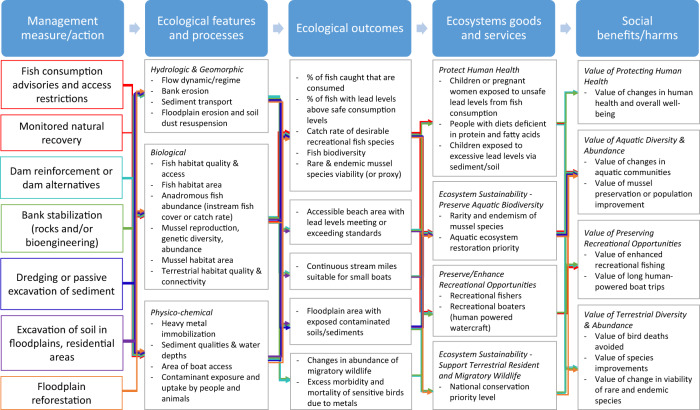
Causal chain model for the Meramec case study. Proposed management actions in the first column are causally linked to ecological features and processes that are expected to change as a result of the restoration. The second column represents indicator categories that are typically used in USACE planning for environmental restoration projects. The third through fifth columns are the elements of the EGS Framework that connect these indicators to increasingly robust benefit measures. Ecological outcomes are indicators of biophysical changes that communicate importance of changes to a non-technical audience. The Ecosystem goods and services column identifies expected beneficiaries or conservation priorities. The Social benefits/harms column suggests indicators of economic value for evaluating projects. Colored arrows represent distinct connection pathways between model elements but many-to-many connections are common

The EGS Framework adds high-level conceptual modeling of all EGS effects to explicitly evaluate who is likely to benefit from biophysical changes created from management actions. This step has no analogue in current USACE planning, although ecosystem (biophysical) conceptual models are sometimes developed (Ogden et al. 2005; Fischenich 2008; Sparks et al. 2012), but they do not represent expected benefits, as defined here. A causal chain conceptual model was developed for the case study to connect groups of proposed actions from Step 1.1 to potential EGS benefits (Fig. 5). Moving from left to right in Fig. 5, the potential project actions are traced through the cause-and-effect relationships to identify initial biophysical changes, ecological outcomes, potential beneficiaries, and finally social benefits. The EGS potentially affected by the project are shown as major headings in the fourth column of Fig. 5, and beneficiaries (or conservation priorities) are identified below the headings.

The primary goal of creating Fig. 5 is to achieve broad agreement about potential project EGS benefits or harms that may arise from alternative design choices. The rationale for conceptual model choices will only be captured, initially, in meeting notes, as the options are discussed among scientific experts and stakeholders. Subsequent steps add the information and documentation needed for more thorough screening.

Because this was a retrospective analysis of a large project, we had abundant data and information to represent the biophysical effects in the causal chain model. However, translating the basic ecological features and processes (Table 3) into ecological outcomes required consulting multiple technical reports and published studies or making inferences based on our understanding of typical goals. We grouped the EGS into protecting, enhancing or supporting (1) human health, (2) aquatic biodiversity, (3) recreation, and (4) terrestrial wildlife.

As part of conceptual modeling, we created new indicators to evaluate and communicate the relative importance or significance of the ecological features and processes to non-technical audiences. For example, the USACE goal to “…protect and restore degraded aquatic and freshwater mussel habitat” (USACE St. Louis District 2019) was connected to an ecological outcome indicator of rare and endemic mussel species viability (Fig. 5, Column 3). Then, the ecological outcomes were connected to an indicator representing degree of stakeholder concern, *rarity and endemism of mussel species* and *aquatic ecosystem restoration priority* (Fig. 5, Column 4). The rare and endemic mussel species viability indicator differs from current USACE guidance by using a population level (persistent) effect on species to be consistent with how people are likely to value improvements in species (e.g., Richardson and Loomis 2009). In contrast, current USACE methods are to apply Habitat Suitability Indices (HSIs) to measure any level of species improvements on a 0–1 scale representing habitat quality for all life cycle events.

In developing the causal chain model, we found that highly protective regulatory requirements for toxic contaminants may not be well-captured by indicators measuring the likely number of beneficiaries. For EPA, “The National Contingency Plan (NCP, 40 C.F.R. Part 300), requires the EPA to evaluate remedial alternatives against nine criteria to determine which alternative is preferred.” (US EPA Region 7 2018, p. 38). In the case study, the level of human lead exposure was generally low, though it reached a potentially harmful level in children eating fish (with high uncertainty) or children with long-duration exposure at a contaminated beach. The number of children meeting these criteria, under the baseline scenario, appears small and might not be interpreted as a substantial effect using the framework methods to count beneficiaries.

The process of creating the conceptual model forces assumptions to be made explicit so that they can be evaluated by the full planning team and stakeholders. The new information required for the causal chain modeling, relative to current guidance, includes evidence for meaningful ecological outcomes and some evidence for the degree of human use or concern for primary and ancillary EGS. Meaningful ecological outcomes are changes that can sufficiently overcome environmental stressors. For example, the project planners used reduced lead ingestion by rodents as a benefit indicator for protection of bird species. In contrast, the framework guides users to consider the relative influence of all lead sources, including lead shot, which is not being controlled by the project, when evaluating improvements to bird populations. Another difference from current planning is that the framework recommends applying a comprehensive list of EGS to screen project elements, as a reminder to consider preferences of and any conflicts among different types of users.

### Step 1.3 Rate EGS Impacts and Examine EGS Planning Relevance

In the EGS Framework, the conceptual model developed in Step 1.2 is used in Step 1.3 to create an Impact Evaluation Table (Table 4) to rapidly summarize the project’s potential EGS outcomes and benefits. The table summarizes the magnitude of biophysical changes, magnitude of potential benefits, and, finally, scarcity and substitutability of EGS to generate a relative measure of outcome importance or value per EGS. This rapid benefit screening differs from current planning in that evidence, uncertainties, and assumptions about potential benefits or harms that are used to create an initial set of project alternatives are made explicit. Further, all potential EGS benefits are evaluated for social benefits, regardless of whether they are within missions and project authorities. Project actions and EGS impacts are further screened using a Decision Criteria Table to reveal constraints on federal cost-sharing and compliance with federal laws, regulations, and relevant executive orders (example in Wainger et al. 2020).

**Table 4:** Illustrative impact evaluation table for Meramec case study (combined EPA and USACE actions)^a^

EGS	Biophysical			Beneficiary			Substitutability	Aggregate Impact Rating
Major goals	Magnitude +/− small positive or negative change; ++/−− medium change; +++/−−− large change; o no / negligible change; ii insufficient information	Duration *temporary; **short term; ***long term	Substantial Impact? See Table 2 † = low confidence	Magnitude +/− small positive or negative effect; ++/−− medium effect; +++/−−− large effect; o no / negligible effect ii insufficient information	Duration *temporary; **short term; ***long term	Substantial Impact? See Table 3 † = low confidence	low, medium, high	See Fig. 3 () = negative † = low confidence
**Protect Human Health** Children or pregnant women exposed to unsafe lead levels from fish consumption	+	** Natural recovery occurs over long term	No†	+	*** From individual’s perspective	No†	na	Negligible†
**Protect Human Health** People with diets deficient in protein and fatty acids	-	** Natural recovery occurs over long term	No†	-	*** From individual’s perspective	No†	na	(Negligible)†
**Protect Human Health** Children exposed to excessive lead levels via sediments	+	***	No	o Risk is to small # of “maximally exposed individuals”	*** From individual’s perspective	No	na	Negligible
**Ecosystem Sustainability** Preserve Aquatic Biodiversity Endangered/endemic mussels	++ Toxicant control positive; Genetic diversity possibly negative. Toxicant effect thought to be most limiting on mussel health.	***	Yes	+++ Highly endangered species (G1) and site is a high national preservation priority	***	Yes†	Low	High
**Ecosystem Sustainability** Enhance Aquatic Habitat Fish abundance & diversity	+	***	Yes	++ Common aquatic species but abundance supports mussel reproduction & habitat quality; Moderate restoration priority	***	No	Med	Med
Ancillary benefits								
**Preserve/Enhance Recreational Opportunities** Recreational fishing (via game fish abundance)	+ Dams and riffles have mixed effects but project expected to improve fish abundance over long term	***	Yes†	++ Site is potentially heavily used	***	Yes	Low-Med	Med†
**Preserve/Enhance Recreational Opportunities** Recreational boating (open navigation for kayaks, canoes or other human powered watercraft)	– Long trips not supported and rocked shorelines can interfere with boat landing	***	Yes	- Current users have expressed concerns, but total number of affected users is unknown	***	Yes†	Low	(Med)†
**Ecosystem Sustainability** Support (improve condition of) terrestrial resident and migratory wildlife	+	***	No† Other Pb sources (shot) not well controlled	o Not a national priority	***	No†	High	Low†

^a^Results for a hypothetical project option containing the following: Fish consumption advisories, reinforce dams & add riffle, targeted excavation of streambeds and floodplains, shoreline stabilization with rocks, sediment traps & trees

#### Impact assessment

An Impact Evaluation Table (Table 4) was completed for a single hypothetical project alternative in which a subset of management actions was used. This hypothetical alternative being represented in Table 4 is generally consistent with the preferred plans selected by US EPA and USACE but adds project elements from earlier phases conducted by US EPA for completeness. Actions include (1) fish consumption advisories, (2) reinforce dams & add riffle, (3) targeted excavation of streambeds and floodplains, and (4) shoreline stabilization with rocks, sediment traps & trees. A separate impact table would be created for each alternative, to represent the outcomes of each bundle of actions.

The Impact Evaluation Table reveals the EGS with the highest potential for enhancement and any substantial harms that need to be avoided, minimized or mitigated. Following the scoring methods and results in Table 4, we conclude that the major EGS benefits generated by this project alternative are two aspects of *Ecosystem Sustainability, Preserve Aquatic Biodiversity and Enhance Aquatic Habitat*. The other major EGS benefit appears to be *Recreational Fishing*, which was not used in the USACE planning process. The table also reveals that this set of actions has some potential for negative impacts to health (via diet changes) and recreational boating, but the magnitudes of effects are uncertain, due to insufficient data.

Once Impact Evaluation Tables for each alternative are completed, the set of aggregate impact ratings by EGS is compared (Table 5; details in Wainger et al. 2020). By comparing where EGS magnitudes vary across the columns of Table 5, the decision maker can see how each alternative (bundles of specific project elements) generate tradeoffs and opportunity costs among EGS. This comparison of alternative scenarios differs from USACE planning outputs in terms of how benefits are represented. The USACE produced a table that scored each alternative for Effectiveness, Efficiency, Acceptability and Completeness (e.g., USACE St. Louis District 2019, Tables 3–4) using metrics that generally represent habitat structures or processes (e.g., sediment migration, natural channel), rather than ecological outcomes or benefits. The closest the USACE table comes to an EGS outcomes metric is “Increases Riparian Habitat,” but it differs from the Impact Evaluation Table by not communicating the potential magnitude of effects and levels of human concern.

**Table 5:** Illustrative alternatives comparison – final product of planning Step 1

	Impact Rating		
	Alternative A	Alternative B	Alternative C
Primary Benefits			
**Protect Human Health** Children or pregnant women exposed to unsafe lead levels from fish consumption	Low	Low	Low
**Protect Human Health** People with diets deficient in protein and fatty acids	(Low)†	(Low)†	—
**Protect Human Health** Children exposed to excessive lead levels via sediments	Low	Low	Low
**Ecosystem Sustainability** Preserve Aquatic Biodiversity & Endangered/endemic mussels	Med-High	Med-High	High
**Ecosystem Sustainability** Enhance Aquatic Habitat & Fish abundance & diversity	Med	Med	High
Ancillary benefits			
**Preserve/Enhance Recreational Opportunities** Recreational fishing (via game fish abundance)	Med	High	Med
**Preserve/Enhance Recreational Opportunities** Recreational boating (open navigation for kayaks, canoes, or other human powered watercraft)	(Med)†	(Med)†	—
**Ecosystem Sustainability** Support (improve condition of) terrestrial resident and migratory wildlife	Low	Low	Low

† = low confidence

() = negative effect

- = no or negligible effect

#### Goal compatibility assessment

Potential conflicts among EGS were compared using a goal compatibility chart that evaluated effects of taking each individual proposed action relative to taking no action. In Table 6, the columns represent EGS goals and rows represent proposed management actions. Compatibility between each goal and action are represented by filled or unfilled circles. Filled (black) circles indicate full compatibility, defined as all outcomes of the action will help achieve that goal. Empty circles represent partial or full incompatibility between the action and the goal. Size of the circle represents degree of influence of action on EGS. Temporary effects are ignored.

**Table 6:**
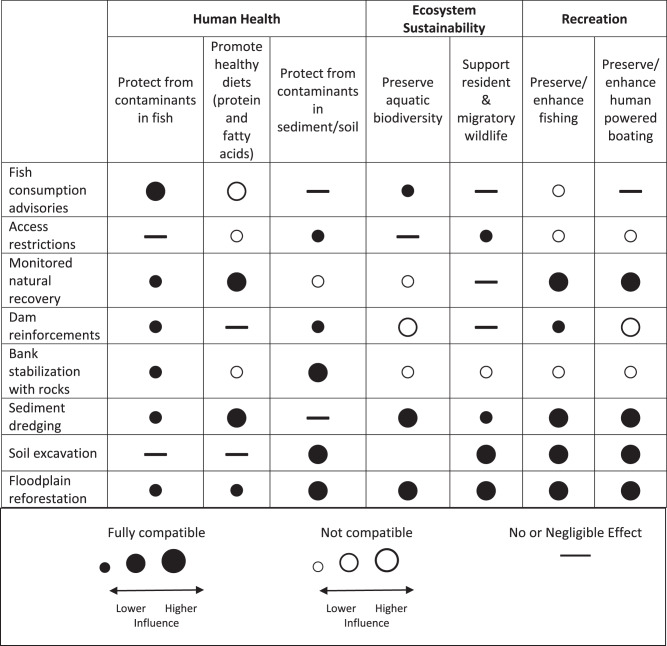
Compatibility of project actions and ecosystem goods and services

Table 6 does not fully represent management bundles since some incompatibilities can be managed by combining actions (in different rows). Also, the table omits opportunity costs, or benefits foregone, of choosing one action over another. Those costs emerge by evaluating whether the maximum benefits for a given EGS are achieved by a project alternative or bundle of project actions (Table 5). An example opportunity cost is that if rocks are chosen to maximize bank stabilization, riparian habitat is diminished.

The primary incompatibilities among EGS identified using Table 6 were that actions to reduce risk from contaminants could conflict with ecosystem sustainability and recreational goals (Table 6). Only three actions were fully compatible across all goals: sediment dredging, soil excavation, and floodplain reforestation. However, sediment dredging and soil excavation had opportunity costs since they did not provide benefits for some EGS and had ecological risks. In contrast, fish consumption advisories, access restrictions, monitored natural recovery, dam reinforcements (or dam alternatives), and bank stabilization with rocks, all had more than one incompatibility, although many were small and potentially manageable with modifications (USACE St. Louis District 2019 Appendix A, p. A-12). Dam reinforcements were seen to have negative effects on recreational boating (human powered) by presenting safety hazards and requiring portage. Also, dam design cannot be simultaneously optimized to prevent sediment movement and to promote fish passage (discussed in USACE St. Louis District 2019 Appendix A.). Finally, an indirect effect being captured is that fish consumption advisories, if successful in reducing fish consumption, have the potential to reduce consumption of healthy fatty acids and proteins (Ginsberg and Toal 2009) among populations that have negligible risk from contaminant levels (Engelberth et al. 2013).

## EGS Framework Discussion

In this discussion, we apply findings from the case study and other projects with which we have been involved to address the questions raised in the introduction about whether the EGS Framework is likely to be worth the effort to apply. Since we only evaluated the project scoping steps, we cannot explicitly compare net benefits for the actual vs hypothetical planning methods. However, we can evaluate the likely costs of application and the thoroughness with which EGS benefits were included in order to examine likely implications for altered decisions and project benefits.

### Does EGS Planning Cost More than Current USACE Planning?

The magnitude of cost increases in the first planning step appeared small for the case study, despite the Framework asking for a broader and more thorough assessment of all expected EGS benefits. We found that the data needed to fill in Table 4 (Impact Evaluation Table) were largely available using reports conducted as part of project planning, albeit with gaps in assessing beneficiary effects and large uncertainties in multiple metrics that are common during initial project scoping. However, small projects that lack comparably thorough background studies will have less information for assessing EGS impacts.

Scoping costs could increase substantially if new in-depth analyses were needed to address the EGS Framework requirement for benefit-relevant ecological indicators or beneficiary effects. Ecological indicators that reflect human concerns can be more costly to evaluate in the field than basic biophysical indicators. For example, fish species richness and catch per unit effort were collected for this case study (Dodd and Wahl 2006) but may not be routinely collected or analyzed. The benefit of this more comprehensive ecological indicator is that it has been linked to human well-being and therefore strongly suggests public benefits (Richardson and Loomis 2009) and may be monetizable with existing economic models (e.g., Johnston and Wainger 2015).

Although filling data gaps can be expensive, low cost methods of using expert judgment or conducting simple analyses with readily available data can manage costs. For example, the number of potential beneficiaries and the scarcity of substitutes can often be addressed with relatively simple Geographic Information System (GIS) analyses of available geospatial data on population. Uncertain information for project comparisons may be more acceptable if monitoring-informed adaptive management is used as part of implementation (as proposed for the case study in US EPA Region 7 2018).

A subtler difference of using the Framework that could increase analytic costs is that the scope of some analyses would need to be broadened to fully characterize benefits. For example, EPA health risk analyses usefully characterized the degree to which people would benefit (avoid morbidity and mortality) from management actions (US EPA Region 7 2018), but did not model a full enough set of behavioral responses to understand net health benefits (see Table 4). Similarly, the reduction in risk to bird populations from lead contamination through contaminated prey species suggested a positive effect on birds, but the net effect was uncertain because other probable pathways of lead ingestion (e.g., lead shot) were omitted. Finally, although not a problem for the case study, some projects will incur costs from documenting the importance of habitat restoration because restoration priorities have not been established for all project sites, and doing so has been identified as a need (Sagoff 1998; Noss et al. 2009; Akçakaya et al. 2018; IUCN SSC 2021).

### Are EGS Benefits Likely to Increase as a Result of the EGS Framework Implementation?

Any answers to this question are highly speculative since we are not able to directly compare decisions made using alternative frameworks. Further, we know that laws, policies and processes of the USACE and US EPA require that some EGS be given more emphasis than others in decision making (e.g., Shabman 2019). However, we can imagine likely outcomes if such constraints were lifted and the decision support process was effective for weighing all potential EGS benefits and tradeoffs. The systematic and comprehensive methods of the EGS Framework suggest that it would provide more relevant information for maximizing diverse benefits, compared to a process that maximizes a small set of benefits or minimizes specific harms (e.g., from contaminant exposure).

Data gaps about beneficiary effects created uncertainties in the case study about whether specific EGS impacts were significant. The Meramec/Big River project documented a high degree of human concern for EGS related to species and ecosystem sustainability (USACE 2019, Tables 1–1), but did not document effects to recreational or other users. If those omitted EGS changes were significant, then different project design choices would have been more likely to maximize comprehensive project benefits.

More generally, the mechanism of comparing tradeoffs among all substantial EGS changes can increase total project benefits, if this awareness is used to adjust project elements to minimize or mitigate EGS impacts. The incompatibilities or opportunity costs of choosing some project elements (Table 6) were not explicitly documented in the original study, suggesting that they were not considered in detail and may have contributed to stakeholder concerns that led to delays late in the project planning process. A specific example is that wetland creation was deemed inefficient for study objectives related to riparian improvements, but the US Fish and Wildlife Service identified benefits of wetlands restoration that might have led to a different conclusion if a multi-benefit perspective was used (USACE St. Louis District 2019 Appendix A. p. A-13). Results from the Impact Evaluation Table (Table 4) also showed some potential for small negative impacts to health (via diet changes) and recreational boating that were not addressed in documentation (USACE St. Louis District 2019). Media reports quoted recreational boaters who said that they were not consulted about measures that interfere with their activities, such as the Newberry Riffle, a short check dam (Chen 2018).

The sequential nature of decision making in which the US EPA first implemented actions to reduce toxic contaminant exposure, before consulting with the USACE on habitat goals, might have limited the magnitude of some project benefits due to EGS incompatibilities. In contrast, the EGS Framework is designed to identify compatibilities among goals across agencies and other stakeholders early in the process, in order to design projects that balance many objectives. Fully realizing the benefits of the framework will likely require meaningful cross-agency collaboration and engagement with local partners, during all planning phases.

### Is Adopting the Framework Likely to Generate Net Benefits?

Although the Framework is likely to add modest time costs to the scoping steps, the real-world outcomes of the case study suggest that these up-front costs might have paid off by preventing the need to make project changes late in the planning process. The document proposing a specific project plan, known as the interim record of decision, was retracted due to unrecognized stakeholder concerns (US EPA Region 7 2019), implying that substantial additional costs will be incurred in redesigning and reanalyzing project alternatives. This outcome suggests that having a framework, such as the EGS Framework, to systematically document all stakeholder concerns at project inception, and clearly showing how they were addressed and why, has some potential to prevent project delays due to unanticipated concerns or as a result of stakeholders perceiving that their concerns were ignored.

More generally, requiring teams to be explicit about their assumptions of how project elements generate benefits and risks to people has the potential to increase net social benefits of projects by improving the information used during design. The conceptual modeling step of the EGS Framework enables the cause and effect relationships that are currently implicit to become explicit so that they can be tested and refined using the knowledge of interdisciplinary and representative teams. The current practice of using basic biophysical indicators to suggest EGS benefits often omits important information that determines whether changes will be substantial and desirable.

Other net benefits are suggested by the potential to increase internal agency planning efficiency. If project teams have clear definitions and procedures for identifying use and nonuse EGS benefits of a project, they can avoid the costly trial and error process of developing EGS analyses that has been used in the past (Cushing et al. 2023). The decision trees and tables in the Framework allow for a consistent means to justify and document the inclusion of EGS across studies. Consistent documentation would allow more efficient internal review at USACE headquarters, since information will have more consistent organization and presentation methods. Other time savings could be realized if project scoping steps are used to generate outputs appropriate for NEPA reporting. The EGS Framework’s requirement to characterize benefits and harms as ‘substantial’ has parallels to NEPA requirements for assessing significant/ insignificant effects (USACE 2020).

We have framed potential benefits in terms of a multi-benefit analysis in which all EGS benefits are valued. Concerns about this approach were raised by USACE planners who suggested that it could put species and habitat restoration goals at risk if EGS encompassing human uses (e.g., recreation) were given high weight during project design. This concern is valid when the project elements needed to produce some EGS benefits have negative effects on nonuse EGS (i.e., harm to species or ecosystems). In the case study, certain types of dam rebuilding or reinforcement could put aquatic biodiversity goals at risk (Table 6). However, the EGS Framework is a decision support tool and does not require that all EGS benefits be given equal weight in project formulation. Subsequent steps in the Framework call for the application of multi-criteria decision analysis to weight the EGS changes by the degree of management concern and alignment with authorities of involved agencies. Revealing the tradeoffs is meant to inform project design choices (e.g., alternative structures to prevent sediment migration) but not preclude achieving the primary project goals.

## Conclusions

Overall, our work suggests that embracing the application of an EGS Framework in project planning has the potential to increase net benefits by reducing planning costs that could emerge from overlooking stakeholder concerns that fall outside of project authorities or regulation. We used a case study to suggest that the modest cost increases associated with a comprehensive EGS scoping analysis may be offset by reduced planning costs if early and thorough evaluation of stakeholder concerns had prevented the need for a project re-design near the end of the planning process. Further, we conclude that projects are more likely to use resources efficiently if the benefits of management measures can be objectively assessed in terms of magnitude of social benefits. Existing methods for choosing environmental restoration rely on biophysical measures with uncertain relationships to human well-being. Improved benefit assessment will come from incorporating multiple sources of risk and human behavioral responses to management measures to improve estimates of the magnitude of EGS change due to a project. However, analysis alone does not ensure change. Effective use of the information produced by any EGS Framework will require some procedural changes and cooperation across government and non-governmental entities in order to realize the benefits.

## Data Availability

All cited reports are publicly available. No original data were created for this project.
